# Crystal structure of phenyl 2,4,5-tri­chloro­benzene­sulfonate

**DOI:** 10.1107/S2056989016007325

**Published:** 2016-05-06

**Authors:** Sean Riley, Richard J. Staples, Shannon M. Biros, Felix N. Ngassa

**Affiliations:** a Department of Chemistry, Grand Valley State University, 1 Campus Dr., Allendale, MI 49401, USA; b Center for Crystallographic Research, Department of Chemistry, Michigan State University, 578 S. Shaw Lane, East Lansing, MI, 48824, USA

**Keywords:** crystal structure, sulfonate, C—Cl⋯π inter­actions

## Abstract

The title compound, C_12_H_7_Cl_3_O_3_S, was synthesized *via* a nucleophilic substitution reaction between phenol and 2,4,5-tri­chloro­benzene­sulfonyl chloride. The two aryl rings are oriented *gauche* to one another around the sulfonate S—O bond, with a C—S—O—C torsion angle of −70.68 (16)°, and the two rings are inclined to one another by 72.40 (7)°. In the crystal, mol­ecules are linked *via* various C—Cl⋯π inter­actions, forming ribbons propagating along [100]. Neighboring ribbons are linked by a weak C—Cl⋯π inter­action, forming layers parallel to (010).

## Abstract 2

In the title compound, the two aryl rings are oriented *gauche* to one another, around the sulfonate S—O bond, with a dihedral angle of 72.40 (7)°. In the crystal, mol­ecules are linked *via* C—Cl⋯π inter­actions, forming ribbons along the *a*-axis direction.

## Chemical context   

The use of arene-sulfonates as leaving groups has been explored in synthetic organic chemistry for quite some time (Crossland *et al.*, 1971 ▸; Klán *et al.*, 2013 ▸; Sardzinski *et al.*, 2015 ▸). The stability of sulfonate ester leaving groups and the identi­fication of suitable protecting groups for sulfonates has been reported (Miller, 2010 ▸). A competitive C—O and S—O bond fission has been reported in the reaction of amine nucleophiles with arene-sulfonates (Um *et al.*, 2004 ▸). The basicity of the amine nucleophile and the electronic nature of the substituent on the sulfonyl moiety are responsible for the difference in regioselectivity. We have synthesized various arene-sulfonate analogues in order to investigate the factors responsible for the competition between C—O and S—O bond fission in the reaction with nitro­gen nucleophiles (Atanasova *et al.*, 2015 ▸; Cooley *et al.*, 2015 ▸).
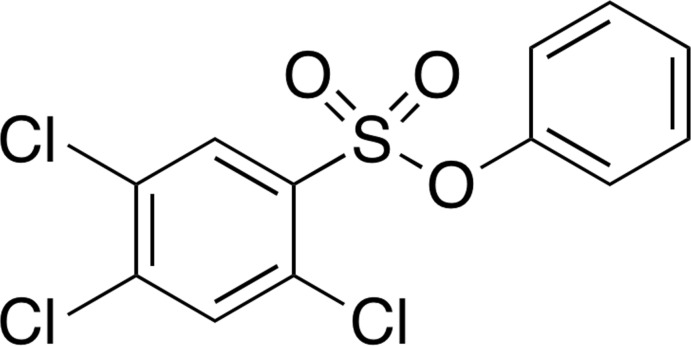


The sulfonamide moiety has found many useful applications in medicinal chemistry (Navia, 2000 ▸). Sulfonamides can be synthesized conveniently from the corresponding sulfonyl chloride and amine nucleophiles. In our recent work, we reported on the synthesis and crystal structure of a chiral sulfonamide (Ngassa *et al.*, 2015 ▸). The direct synthesis of sulfonamides from arene-sulfonates has been reported (Caddick *et al.*, 2004 ▸). Taking advantage of the regioselectivity of C—O *vs* S—O bond fission, we have explored the use of arene-sulfonates as electrophilic substrates in the synthesis of sulfonamides. We are inter­ested in the role of the substituent on the sulfonyl moiety and the basicity of the amine nucleophile on the nucleophilic substitution. As the title compound is of inter­est in our ongoing effort to investigate the role of the substituent on the sulfonyl moiety in nucleophilic substitution reactions with nitro­gen- and oxygen-nucleophiles, we report herein on the synthesis and crystal structure of this electrophilic arene-sulfonate.

## Structural commentary   

**Figure 1:**
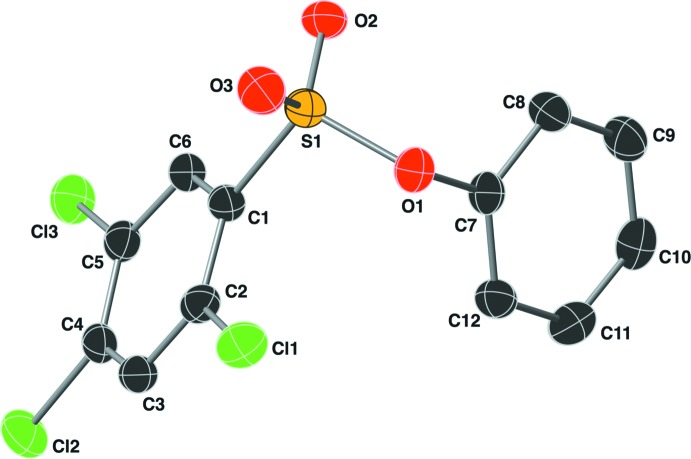
The mol­ecular structure of the title compound, showing the atom labeling. Displacement ellipsoids are drawn at the 50% probability level.

The mol­ecular structure of the title compound is shown in Fig. 1 ▸. The two aryl rings are oriented gauche to one another around the sulfonate S1—O1 bond, with a C1—S1—O1—C7 torsion angle of −70.68 (16)°. The two rings (C1–C6 and C7–C12) are inclined to one another by 72.40 (7)°.

## Supra­molecular features   

**Figure 2:**
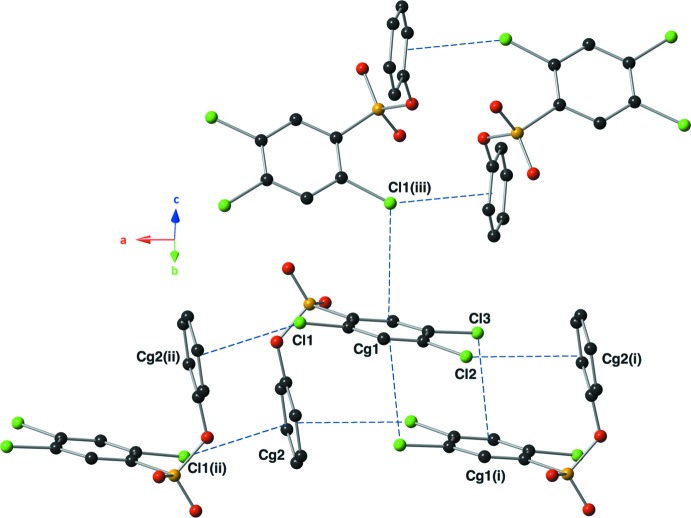
A view of the various C—Cl⋯π inter­actions (blue dashed lines; see Table 1 ▸) present in the crystal lattice of the title compound. H atoms have been omitted for clarity [symmetry codes: (i) −*x* + 2, −*y* + 1, −*z* + 1; (ii) −*x* + 1, −*y* + 2, −*z* + 1; (iii) −*x* + 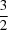, *y* + 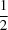, −*z* + 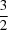].

In the crystal, mol­ecules are linked by Cl⋯π inter­actions (Table 1 ▸ and Fig. 2 ▸). These inter­molecular inter­actions range in Cl⋯ring centroid distances from 3.525 (1) to 3.972 (1) Å (Table 1 ▸). This distance falls near the accepted average as previously noted (Imai, *et al.*, 2008 ▸), and all inter­actions have a ‘face-on’ geometry. The two strong inter­actions involving atoms Cl1 and Cl2 with the centroid of ring C7–C12 form ribbons propagating along the *a-*axis direction. Within the ribbon there is also a weaker Cl⋯π inter­action involving atom Cl3 and the centroid of ring C1–C6. Neighbouring ribbons are linked by a second weak Cl1⋯π inter­action (Table 1 ▸ and Fig. 2 ▸), forming layers parallel to the *ac* plane. There are no other significant inter­molecular inter­actions present in the crystal.

## Database survey   

The Cambridge Structural Database (CSD, Version 5.37, February 2016; Groom *et al.*, 2016 ▸) contains eight structures of phenyl sulfonates where the group bonded directly to the sulfur atom is an aromatic ring. Other substituents on this ring include *p*-tolyl (FIQCIS: Manivannan *et al.*, 2005 ▸), nitro (AJIWUL: Vembu *et al.*, 2003 ▸; XUKBOV: Vembu & Fronczek, 2009 ▸), napthyl (VOJBOM: Vennila *et al.*, 2008 ▸) and amino-napthyl (LEZWAP: Beyeh *et al.*, 2007 ▸). Of particular inter­est is the structure JEGWEY (Wright *et al.*, 2006 ▸) where the substituted aromatic ring bears chlorine atoms in the 2- and 5-positions. The torsion angle around the sulfonate S—O bond is 73.15 (19)°, similar to that seen in the title compound [70.68 (16)°]. In the crystal of this compound, one C—Cl⋯π inter­action is present [Cl⋯π distance: 3.4187 (16) Å] along with C—H⋯O hydrogen bonds.

Two recent publications describing the crystal structures of benzopyrimidoazepine derivatives have also noted C—Cl⋯π inter­actions present in the lattice (Acosta *et al.*, 2015 ▸; Acosta Qu­intero *et al.*, 2016 ▸). In these examples, the C—Cl⋯π inter­actions are complemented by either C—H⋯π or π–π inter­actions between mol­ecules in the solid state.

## Synthesis and crystallization   

Phenol (0.941g, 10 mmol) was dissolved in 10 ml of chilled di­chloro­methane. This was followed by the addition of pyridine (1.6 ml, 20 mmol). The resulting solution was cooled in an ice bath under an N_2_ atmosphere, followed by the addition of 2,4,5-tri­chloro­benzene­sulfonyl chloride (1.91 g, 10 mmol) portion-wise. The mixture was stirred at 273 K for 30 min and then at room temperature for 12 h. Reaction completion was verified by using TLC analysis. After dilution with 15 ml of CH_2_Cl_2_, the organic phase was washed with H_2_O, brine, and dried over anhydrous Na_2_SO_4_. After the solvent was evaporated the crude product was obtained as a tan solid. The title compound was recrystallized from CH_2_Cl_2_/hexa­nes to afford colourless needle-like crystals (56% yield, m.p. 380–381 K) suitable for X-ray diffraction analysis.

## Refinement   

Crystal data, data collection and structure refinement details are summarized in Table 2 ▸. The positions of all hydrogen atoms were calculated geometrically and refined to ride on their parent atoms: C—H = 0. 95 Å with *U*_iso_(H) = 1.2*U*_eq_(C).

## Supplementary Material

Crystal structure: contains datablock(s) global, I. DOI: 10.1107/S2056989016007325/su5297sup1.cif

Structure factors: contains datablock(s) I. DOI: 10.1107/S2056989016007325/su5297Isup2.hkl

Supporting information file. DOI: 10.1107/S2056989016007325/su5297Isup3.cml

CCDC reference: 1477649

Additional supporting information:  crystallographic information; 3D view; checkCIF report

## Figures and Tables

**Table 1:** Geometric parameters (Å, °) for C—Cl⋯π contacts in the title compound. *Cg* 1 and *Cg*2 are the centroids of rings C1–C6 and C7–C12, respectively.

C—Cl⋯*Cg*	C—Cl	Cl⋯*Cg*	C⋯*Cg*	C—Cl⋯*Cg*
C2—Cl1⋯*Cg*2^i^	1.727 (2)	3.5250 (10)	5.028 (2)	144.23 (7)
C4—Cl2⋯*Cg*2^ii^	1.721 (2)	3.7914 (11)	5.160 (2)	135.37 (7)
C5—Cl3⋯*Cg*1^ii^	1.725 (2)	3.6298 (10)	4.211 (2)	97.25 (7)
C2—Cl1..*Cg*1^iii^	1.727 (2)	3.9722 (10)	4.989 (2)	116.56 (7)

Symmetry codes:(i) −*x* + 2, −*y* + 1, −*z* + 1; (ii) −*x* + 1, −*y* + 2, −*z* + 1; (iii) −*x* + 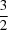, *y* + 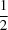, −*z* + 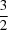.

**Table 2:** Experimental details

Crystal data	
Chemical formula	C_12_H_7_Cl_3_O_3_S
*M*_r_	337.59
Crystal system, space group	Monoclinic, *P*2_1_/*n*
Temperature (K)	173
*a*, *b*, *c* (Å)	12.3401 (11), 6.5421 (6), 16.1350 (14)
β (°)	92.1159 (10)
*V* (Å^3^)	1301.7 (2)
*Z*	4
Radiation type	Mo *K*α
μ (mm^−1^)	0.86
Crystal size (mm)	0.24 × 0.18 × 0.10

Data collection	
Diffractometer	Bruker APEXII CCD
Absorption correction	Multi-scan (*SADABS*; Bruker, 2013 ▸)
*T*_min_, *T*_max_	0.689, 0.745
No. of measured, independent and observed [*I* > 2σ(*I*)] reflections	10912, 2568, 2172
*R*_int_	0.029
(sin θ/λ)_max_ (Å^−1^)	0.618

Refinement	
*R*[*F* ^2^ > 2σ(*F* ^2^)], *wR*(*F* ^2^), *S*	0.031, 0.083, 1.06
No. of reflections	2568
No. of parameters	172
H-atom treatment	H-atom parameters constrained
Δρ_max_, Δρ_min_ (e Å^−3^)	0.26, −0.28

Computer programs: *APEX2* (Bruker, 2013 ▸), *SAINT* (Bruker, 2013 ▸), *SHELXS2014* (Sheldrick, 2008 ▸), *SHELXL2014* (Sheldrick, 2015 ▸), *OLEX2* (Dolomanov *et al.*, 2009 ▸; Bourhis *et al.*, 2015 ▸), *CrystalMaker* (Palmer, 2007 ▸).

## supplementary crystallographic information

### Crystal data

**Table:**

C_12_H_7_Cl_3_O_3_S	*F*(000) = 680
*M**_r_* = 337.59	*D*_x_ = 1.723 Mg m^−^^3^
Monoclinic, *P*2_1_/*n*	Mo *K*α radiation, λ = 0.71073 Å
*a* = 12.3401 (11) Å	Cell parameters from 5969 reflections
*b* = 6.5421 (6) Å	θ = 2.5–26.0°
*c* = 16.1350 (14) Å	µ = 0.86 mm^−^^1^
β = 92.1159 (10)°	*T* = 173 K
*V* = 1301.7 (2) Å^3^	Needle, colourless
*Z* = 4	0.24 × 0.18 × 0.10 mm

### Data collection

**Table:**

Bruker APEXII CCD diffractometer	2172 reflections with *I* > 2σ(*I*)
φ and ω scans	*R*_int_ = 0.029
Absorption correction: multi-scan (*SADABS*; Bruker, 2013)	θ_max_ = 26.1°, θ_min_ = 2.0°
*T*_min_ = 0.689, *T*_max_ = 0.745	*h* = −15→15
10912 measured reflections	*k* = −8→8
2568 independent reflections	*l* = −19→19

### Refinement

**Table:**

Refinement on *F*^2^	0 restraints
Least-squares matrix: full	Hydrogen site location: inferred from neighbouring sites
*R*[*F*^2^ > 2σ(*F*^2^)] = 0.031	H-atom parameters constrained
*wR*(*F*^2^) = 0.083	*w* = 1/[σ^2^(*F*_o_^2^) + (0.0396*P*)^2^ + 0.7507*P*] where *P* = (*F*_o_^2^ + 2*F*_c_^2^)/3
*S* = 1.06	(Δ/σ)_max_ = 0.001
2568 reflections	Δρ_max_ = 0.26 e Å^−^^3^
172 parameters	Δρ_min_ = −0.28 e Å^−^^3^

### Special details

**Table:**

Geometry. All esds (except the esd in the dihedral angle between two l.s. planes) are estimated using the full covariance matrix. The cell esds are taken into account individually in the estimation of esds in distances, angles and torsion angles; correlations between esds in cell parameters are only used when they are defined by crystal symmetry. An approximate (isotropic) treatment of cell esds is used for estimating esds involving l.s. planes.

### Fractional atomic coordinates and isotropic or equivalent isotropic displacement parameters (Å^2^)

**Table:**

	*x*	*y*	*z*	*U*_iso_*/*U*_eq_	
Cl3	0.40330 (4)	0.76082 (9)	0.56242 (3)	0.03498 (15)	
Cl2	0.43839 (5)	1.20119 (9)	0.64173 (4)	0.04126 (17)	
Cl1	0.86246 (4)	1.05869 (8)	0.68164 (3)	0.03599 (16)	
S1	0.83040 (4)	0.61521 (8)	0.58959 (3)	0.02676 (14)	
O2	0.78797 (12)	0.4493 (2)	0.54149 (9)	0.0322 (3)	
O3	0.88861 (13)	0.5784 (2)	0.66557 (9)	0.0367 (4)	
C1	0.72261 (16)	0.7865 (3)	0.60570 (12)	0.0250 (4)	
C12	0.86103 (16)	0.9951 (3)	0.43502 (13)	0.0297 (5)	
H12	0.8568	1.0940	0.4779	0.036*	
O1	0.91219 (11)	0.7437 (2)	0.53709 (8)	0.0283 (3)	
C3	0.64884 (17)	1.1015 (3)	0.65532 (12)	0.0287 (5)	
H3	0.6587	1.2317	0.6806	0.034*	
C6	0.61918 (16)	0.7233 (3)	0.58012 (12)	0.0260 (4)	
H6	0.6093	0.5947	0.5535	0.031*	
C8	0.89316 (17)	0.6461 (3)	0.39295 (13)	0.0309 (5)	
H8	0.9107	0.5088	0.4072	0.037*	
C5	0.53069 (16)	0.8467 (3)	0.59320 (12)	0.0267 (4)	
C4	0.54605 (17)	1.0382 (3)	0.62926 (12)	0.0285 (5)	
C2	0.73697 (17)	0.9755 (3)	0.64467 (12)	0.0273 (4)	
C7	0.88520 (16)	0.7940 (3)	0.45289 (12)	0.0256 (4)	
C10	0.84980 (18)	0.9035 (4)	0.29115 (13)	0.0352 (5)	
H10	0.8371	0.9415	0.2348	0.042*	
C11	0.84316 (18)	1.0485 (3)	0.35269 (14)	0.0345 (5)	
H11	0.8262	1.1860	0.3385	0.041*	
C9	0.87469 (18)	0.7041 (4)	0.31090 (14)	0.0359 (5)	
H9	0.8793	0.6053	0.2680	0.043*	

### Atomic displacement parameters (Å^2^)

**Table:**

	*U*^11^	*U*^22^	*U*^33^	*U*^12^	*U*^13^	*U*^23^
Cl3	0.0269 (3)	0.0401 (3)	0.0379 (3)	0.0003 (2)	0.0017 (2)	0.0008 (2)
Cl2	0.0432 (3)	0.0361 (3)	0.0451 (3)	0.0146 (2)	0.0111 (3)	0.0001 (2)
Cl1	0.0383 (3)	0.0317 (3)	0.0375 (3)	−0.0058 (2)	−0.0056 (2)	−0.0032 (2)
S1	0.0288 (3)	0.0241 (3)	0.0275 (3)	0.0027 (2)	0.0018 (2)	0.0006 (2)
O2	0.0345 (8)	0.0237 (8)	0.0386 (8)	−0.0003 (6)	0.0043 (6)	−0.0037 (6)
O3	0.0419 (9)	0.0386 (9)	0.0295 (8)	0.0100 (7)	−0.0021 (7)	0.0046 (7)
C1	0.0287 (11)	0.0228 (10)	0.0235 (10)	0.0032 (8)	0.0033 (8)	0.0015 (8)
C12	0.0290 (11)	0.0256 (11)	0.0347 (11)	−0.0001 (9)	0.0044 (9)	−0.0039 (9)
O1	0.0244 (7)	0.0334 (8)	0.0271 (7)	−0.0018 (6)	0.0002 (6)	0.0006 (6)
C3	0.0420 (12)	0.0232 (10)	0.0210 (10)	0.0007 (9)	0.0046 (9)	0.0001 (8)
C6	0.0296 (11)	0.0245 (10)	0.0241 (10)	0.0000 (8)	0.0042 (8)	0.0001 (8)
C8	0.0299 (11)	0.0287 (11)	0.0344 (11)	0.0034 (9)	0.0048 (9)	−0.0034 (9)
C5	0.0281 (10)	0.0285 (11)	0.0236 (10)	0.0006 (8)	0.0035 (8)	0.0029 (8)
C4	0.0354 (11)	0.0266 (11)	0.0239 (10)	0.0073 (9)	0.0088 (8)	0.0042 (8)
C2	0.0347 (11)	0.0247 (10)	0.0226 (10)	−0.0043 (9)	0.0011 (8)	0.0006 (8)
C7	0.0212 (10)	0.0305 (11)	0.0251 (10)	−0.0006 (8)	0.0022 (8)	0.0005 (8)
C10	0.0323 (12)	0.0458 (14)	0.0275 (11)	−0.0051 (10)	0.0022 (9)	0.0029 (10)
C11	0.0339 (12)	0.0301 (12)	0.0394 (12)	−0.0003 (9)	−0.0001 (10)	0.0066 (10)
C9	0.0371 (12)	0.0393 (13)	0.0316 (11)	−0.0027 (10)	0.0055 (9)	−0.0108 (10)

### Geometric parameters (Å, º)

**Table:**

Cl3—C5	1.725 (2)	C3—C4	1.385 (3)
Cl2—C4	1.721 (2)	C3—C2	1.380 (3)
Cl1—C2	1.727 (2)	C6—H6	0.9500
S1—O2	1.4229 (15)	C6—C5	1.380 (3)
S1—O3	1.4184 (15)	C8—H8	0.9500
S1—C1	1.766 (2)	C8—C7	1.374 (3)
S1—O1	1.5828 (15)	C8—C9	1.388 (3)
C1—C6	1.390 (3)	C5—C4	1.391 (3)
C1—C2	1.395 (3)	C10—H10	0.9500
C12—H12	0.9500	C10—C11	1.378 (3)
C12—C7	1.377 (3)	C10—C9	1.375 (3)
C12—C11	1.383 (3)	C11—H11	0.9500
O1—C7	1.425 (2)	C9—H9	0.9500
C3—H3	0.9500		
			
O2—S1—C1	107.48 (9)	C6—C5—Cl3	118.90 (16)
O2—S1—O1	110.01 (8)	C6—C5—C4	119.61 (19)
O3—S1—O2	120.41 (9)	C4—C5—Cl3	121.47 (16)
O3—S1—C1	109.92 (9)	C3—C4—Cl2	118.78 (16)
O3—S1—O1	103.89 (9)	C3—C4—C5	120.35 (19)
O1—S1—C1	103.92 (9)	C5—C4—Cl2	120.87 (17)
C6—C1—S1	117.13 (15)	C1—C2—Cl1	122.17 (16)
C6—C1—C2	119.83 (19)	C3—C2—Cl1	117.99 (16)
C2—C1—S1	123.01 (16)	C3—C2—C1	119.84 (19)
C7—C12—H12	121.1	C12—C7—O1	117.49 (18)
C7—C12—C11	117.9 (2)	C8—C7—C12	123.09 (19)
C11—C12—H12	121.1	C8—C7—O1	119.19 (18)
C7—O1—S1	120.10 (12)	C11—C10—H10	119.8
C4—C3—H3	120.0	C9—C10—H10	119.8
C2—C3—H3	120.0	C9—C10—C11	120.3 (2)
C2—C3—C4	120.04 (19)	C12—C11—H11	119.8
C1—C6—H6	119.9	C10—C11—C12	120.4 (2)
C5—C6—C1	120.24 (19)	C10—C11—H11	119.8
C5—C6—H6	119.9	C8—C9—H9	119.7
C7—C8—H8	121.1	C10—C9—C8	120.5 (2)
C7—C8—C9	117.7 (2)	C10—C9—H9	119.7
C9—C8—H8	121.1		
			
Cl3—C5—C4—Cl2	−2.0 (2)	C6—C1—C2—Cl1	177.38 (15)
Cl3—C5—C4—C3	178.38 (15)	C6—C1—C2—C3	−2.0 (3)
S1—C1—C6—C5	177.92 (15)	C6—C5—C4—Cl2	176.71 (15)
S1—C1—C2—Cl1	−0.5 (3)	C6—C5—C4—C3	−2.9 (3)
S1—C1—C2—C3	−179.86 (15)	C4—C3—C2—Cl1	−177.78 (15)
S1—O1—C7—C12	109.03 (18)	C4—C3—C2—C1	1.6 (3)
S1—O1—C7—C8	−76.3 (2)	C2—C1—C6—C5	−0.1 (3)
O2—S1—C1—C6	7.64 (18)	C2—C3—C4—Cl2	−178.78 (15)
O2—S1—C1—C2	−174.44 (16)	C2—C3—C4—C5	0.8 (3)
O2—S1—O1—C7	44.13 (16)	C7—C12—C11—C10	−0.1 (3)
O3—S1—C1—C6	−125.09 (16)	C7—C8—C9—C10	0.3 (3)
O3—S1—C1—C2	52.8 (2)	C11—C12—C7—O1	174.98 (18)
O3—S1—O1—C7	174.30 (14)	C11—C12—C7—C8	0.6 (3)
C1—S1—O1—C7	−70.68 (16)	C11—C10—C9—C8	0.2 (3)
C1—C6—C5—Cl3	−178.73 (15)	C9—C8—C7—C12	−0.6 (3)
C1—C6—C5—C4	2.5 (3)	C9—C8—C7—O1	−174.96 (18)
O1—S1—C1—C6	124.24 (15)	C9—C10—C11—C12	−0.2 (3)
O1—S1—C1—C2	−57.85 (18)		
